# Hospital variation in transfusion and infection after cardiac surgery: a cohort study

**DOI:** 10.1186/1741-7015-7-37

**Published:** 2009-07-31

**Authors:** Mary AM Rogers, Neil Blumberg, Sanjay Saint, Kenneth M Langa, Brahmajee K Nallamothu

**Affiliations:** 1 Division of General Medicine, Department of Internal Medicine, University of Michigan, Ann Arbor, Michigan, USA; 2 Patient Safety Enhancement Program, Ann Arbor Veterans Affairs Medical Center and University of Michigan Health System, Ann Arbor, Michigan, USA; 3 Department of Pathology and Laboratory Medicine, University of Rochester Medical Center, Rochester, New York, USA; 4 Health Services Research and Development Center of Excellence, Ann Arbor Veterans Affairs Medical Center, Ann Arbor, Michigan, USA; 5 Institute of Gerontology, University of Michigan, Ann Arbor, Michigan, USA; 6 Division of Cardiovascular Medicine, Department of Internal Medicine, University of Michigan, Ann Arbor, Michigan, USA

## Abstract

**Background:**

Transfusion practices in hospitalised patients are being re-evaluated, in part due to studies indicating adverse effects in patients receiving large quantities of stored blood. Concomitant with this re-examination have been reports showing variability in the use of specific blood components. This investigation was designed to assess hospital variation in blood use and outcomes in cardiac surgery patients.

**Methods:**

We evaluated outcomes in 24,789 Medicare beneficiaries in the state of Michigan, USA who received coronary artery bypass graft surgery from 2003 to 2006. Using a cohort design, patients were followed from hospital admission to assess transfusions, in-hospital infection and mortality, as well as hospital readmission and mortality 30 days after discharge. Multilevel mixed-effects logistic regression was used to calculate the intrahospital correlation coefficient (for 40 hospitals) and compare outcomes by transfusion status.

**Results:**

Overall, 30% (95 CI, 20% to 42%) of the variance in transfusion practices was attributable to hospital site. Allogeneic blood use by hospital ranged from 72.5% to 100% in women and 49.7% to 100% in men. Allogeneic, but not autologous, blood transfusion increased the odds of in-hospital infection 2.0-fold (95% CI 1.6 to 2.5), in-hospital mortality 4.7-fold (95% CI 2.4 to 9.2), 30-day readmission 1.4-fold (95% CI 1.2 to 1.6), and 30-day mortality 2.9-fold (95% CI 1.4 to 6.0) in elective surgeries. Allogeneic transfusion was associated with infections of the genitourinary system, respiratory tract, bloodstream, digestive tract and skin, as well as infection with *Clostridium difficile*. For each 1% increase in hospital transfusion rates, there was a 0.13% increase in predicted infection rates.

**Conclusion:**

Allogeneic blood transfusion was associated with an increased risk of infection at multiple sites, suggesting a system-wide immune response. Hospital variation in transfusion practices after coronary artery bypass grafting was considerable, indicating that quality efforts may be able to influence practice and improve outcomes.

## Background

The practice of transfusion is in transition. Over the past several decades a body of evidence has accumulated that indicates various adverse effects in patients who receive transfusions, particularly with exposure to allogeneic blood (that is, blood received from a genetically dissimilar individual) [1-5]. Effects include, but are not limited to, postoperative pneumonia, sepsis, and mortality [1-5]. The most notable study to date, a randomised controlled trial of liberal versus conservative red blood cell use [6], demonstrated that, for patients who were less acutely ill, mortality was lower in the group receiving fewer transfusions and, for high risk patients, mortality was similar in lower and higher transfusion groups. Recent studies have implicated prolonged storage of blood products as an important factor [7], although investigations of patients' responses to specific stored blood components are ongoing.

Variation in the use of blood components is substantial. Data from patients who received coronary artery bypass graft (CABG) surgery at 32 hospitals in the US (1996 to 2001) showed the maximum variation possible [8]. During the postoperative period, the use of red blood cells, fresh-frozen plasma and platelets ranged from 0% (no patients transfused) to 100% (all patients transfused) in different hospitals. During the intraoperative period, usage ranged from 0% to approximately 70% for red blood cells and 0% to 50% for fresh-frozen plasma and platelets.

Anticipating that practices may have changed within the last 5 years, we designed a cohort study to evaluate the variation of transfusion use in hospitals using more recent data and to assess in-hospital infection, 30-day readmission, and 30-day mortality in patients by transfusion status.

## Methods

### Participants

Subjects were all fee-for-service Medicare beneficiaries (n = 24,789) who received CABG surgery (International Classification of Diseases, Clinical Modification, ninth edition (ICD-9) procedure codes 36.1×) from 2003 to the end of 2006. Only patients 65 years of age or older, who were Michigan residents or received their surgery in a Michigan hospital, were included. In this retrospective cohort study, patients were followed from hospital admission to 30 days after hospital discharge. Inpatient standard analytical files and denominator files were obtained from the Centers for Medicare and Medicaid Services (CMS), and contained information regarding hospitalisations and Medicare enrolment.

There were two main areas of investigation. The first was the evaluation of differences in transfusion use and infection rates in hospitals and to calculate the intraclass (that is, intrahospital) correlation coefficient. The second was the assessment of the relationship between transfusion and patient outcomes. The primary outcome was infection during hospitalisation. Secondary outcomes were death (in-hospital and 30-days post discharge) and readmission to a hospital (for any reason and for reason of infection). For post-discharge outcomes, only those individuals who survived to hospital discharge were included in the analyses. Since infection was the primary outcome, we excluded those patients who were initially admitted for reason of infection (prior to the CABG procedure) and those with evidence of pre-existing infection (for example, acquired immunodeficiency syndrome) during the hospital stay when the CABG procedure occurred. This constituted 0.4% of the sample (n = 115 patients).

### Measures

Data regarding blood transfusions were extracted from procedure codes (99.0×), as well as revenue codes for blood products and services (38× for purchased blood and 39× for donated blood). For purposes of these analyses, the receipt of an allogeneic transfusion could have included any of the following components at any time during hospitalisation: red blood cells, whole blood, platelets, plasma or cryoprecipitates. The use of autologous blood (where donor and recipient were the same individual) was also obtained from two procedure codes (99.00: perioperative autologous transfusion of whole blood or blood components; 99.02: transfusion of previously collected autologous blood).

We determined infection by using ICD-9 codes that explicitly stated infection (for example, 0xx.xx) or provided evidence of infection (purulent, suppurative, septic, pyogenic or abscess). Data were also extracted regarding age, gender, race, secondary diagnoses, type of admission (elective, urgent, emergency), and length of stay. Less than 1% of values for race and type of admission were missing and were imputed using best subset regression. We examined race at both the patient and hospital levels; specifically, for purposes of this investigation, hospitals were classified as African-American if ≥ 50% of the patients who received CABG surgery annually were African-American.

Surgeon volume was determined by summing the number of Medicare CABG procedures per operating physician, calculating the annual mean, and categorising into 2 equal groups based on the median number of cases per year (60 CABG procedures/year). Hospital volume was determined by summing the number of Medicare CABG procedures and calculating the annual mean. We then categorised hospitals into 2 equal groups based on the median number of cases per year (240 CABG procedures/year). For the analyses of hospital measures and intraclass correlation coefficients, the analyses were restricted to those hospitals that performed at least 50 CABG procedures (n = 40 hospitals).

### Statistical analyses

Patient characteristics were evaluated first by receipt of allogeneic blood transfusion. Bivariate associations were assessed using Pearson χ^2 ^tests for categorical data and the Wilcoxon rank sum test for differences in median length of hospital stay. Multilevel mixed-effects logistic regression was used to evaluate the associations between transfusion and study outcomes (in-hospital infection, 30-day readmission, 30-day mortality). A two-level hierarchical model was used in which patients were nested within hospitals. The hospital was modelled as a random intercept with transfusion included as a fixed effect. The structure of the covariance matrix for the random effect was specified using the identity structure (uncorrelated random effects with common variance). In postestimation, predicted probabilities were calculated based on the linear predictor of both fixed and random effects. The intraclass correlation coefficient for the multilevel logistic model was calculated as described by Snijders and Bosker [9].

In order to address the possible confounding effect of comorbid conditions, propensity scores were calculated. Specifically, we estimated the propensity for each person to receive a transfusion in order to address the possibility that recipients of blood transfusion had more underlying illnesses than those not receiving transfusions. The probability of receiving an allogeneic blood transfusion was based on the predicted values generated from logistic regression using the following covariates: age, gender, race, type of admission (elective, urgent, emergency), congestive heart failure, diabetes mellitus, renal failure, hypertension, chronic pulmonary disease, malignancy, peripheral vascular disease, cerebrovascular disease, and myocardial infarction (area under the receiver operating characteristic curve = 0.7368). The scores were categorised into deciles. Mean propensity scores were not different among patients transfused and not transfused within each block. In addition to adjustment for propensity decile, all results controlled for surgeon volume and hospital volume. The α was set at 0.05, and all tests were two-tailed. Stata/SE 10.0 software was used for all analyses (Stata, College Station, TX, USA).

This study was approved by the Institutional Review Board on Human Subjects at the University of Michigan at Ann Arbor and by the Privacy Review Board at CMS.

## Results

### Patient characteristics

Of the 24,789 patients receiving CABG surgery in this cohort, the majority were men (64%), white (92%), and between the ages of 65 and 74 (54%). Table 1 lists the characteristics of the patients. Nearly half of the CABG procedures were performed during elective admissions, and conditions such as diabetes mellitus, hypertension, myocardial infarction, congestive heart failure and chronic pulmonary disease were relatively frequent in this cohort. The median length of hospital stay was 9 days in patients who received a transfusion and 6 days in those who did not (*P *< 0.001).

**Table 1:** Characteristics of patients who underwent coronary artery bypass graft surgery

		Allogeneic transfusion			
				
		Yes	No	Total	
Patient characteristics		n = 20,789	n = 4,000	n = 24,789	*P *value
Age (years):	65 to 69	5,069 (77%)	1,487 (23%)	6,556 (100%)	
	70 to 74	5,488 (82%)	1,238 (18%)	6,726 (100%)	
	75 to 79	5,761 (87%)	876 (13%)	6,637 (100%)	
	80 to 84	3,513 (91%)	350 (9%)	3,863 (100%)	
	≥ 85	958 (95%)	49 (5%)	1,007 (100%)	< 0.001
Gender:	Men	12,522 (78%)	3,438 (22%)	15,960 (100%)	
	Women	8,267 (94%)	562 (6%)	8,829 (100%)	< 0.001
Race:	White	19,035 (83%)	3,804 (17%)	22,839 (100%)	
	Black	1,250 (90%)	133 (10%)	1,383 (100%)	
	Other	504 (89%)	63 (11%)	567 (100%)	< 0.001
Type of admission:	Elective	9,183 (80%)	2,289 (20%)	11,472 (100%)	
	Urgent	6,035 (85%)	1,083 (15%)	7,118 (100%)	
	Emergency	5,571 (90%)	628 (10%)	6,199 (100%)	< 0.001
Hospital CABG volume (annual mean):	< 240	10,988 (87%)	1,595 (13%)	12,583 (100%)	
	≥ 240	9,801 (80%)	2,405 (20%)	12,206 (100%)	< 0.001
Surgeon CABG volume (annual mean):	< 60	10,796 (87%)	1,605 (13%)	12,401 (100%)	
	≥ 60	9,993 (81%)	2,395 (19%)	12,388 (100%)	< 0.001
Coexisting conditions:	Diabetes mellitus	5,747 (82%)	1,227 (18%)	6,974 (100%)	< 0.001
	Renal failure	2,006 (93%)	152 (7%)	2,158 (100%)	< 0.001
	Myocardial infarction	7,207 (86%)	1,157 (14%)	8,364 (100%)	< 0.001
	Congestive heart failure	6,593 (93%)	529 (7%)	7,122 (100%)	< 0.001
	Hypertension	11,063 (80%)	2,851 (20%)	13,914 (100%)	< 0.001
	Peripheral vascular disease	2,491 (84%)	474 (16%)	2,965 (100%)	0.813
	Cerebrovascular disease	1,586 (86%)	265 (14%)	1,851 (100%)	0.027
	Chronic pulmonary disease	5,014 (86%)	830 (14%)	5,844 (100%)	< 0.001
	Malignancy	463 (84%)	85 (16%)	548 (100%)	0.687

CABG = coronary artery bypass graft.

### Transfusion

**Figure 1:**
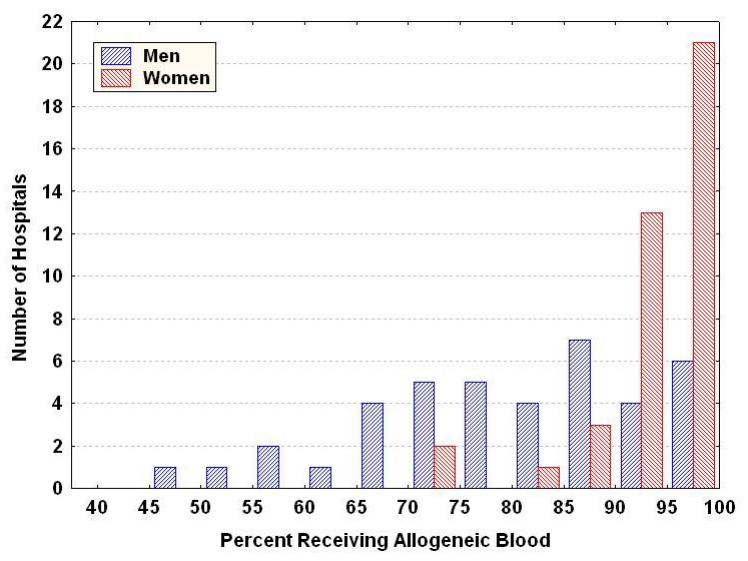
**Variation across hospitals in use of allogeneic blood transfusion for men and women undergoing coronary artery bypass graft surgery**.

Transfusion during hospitalisation was common; allogeneic blood was given to 83.9% of patients and autologous blood (only) was used for 1.2% of patients. Women were more likely to receive a transfusion than men (93.6% vs 78.5%, respectively, for allogeneic blood). There was considerable variation in the use of allogeneic blood across hospitals (Figure 1), ranging from 49.7% to 100% (median 82.2%) in men and from 72.5% to 100% (median 95.7%) in women. Of patients who received a transfusion, the number of units of blood was available for only 1%; in this sample, there was a significant difference in the mean number of units of blood used across hospitals (*P *< 0.0001).

### Infection

Of the 24,789 patients in the study, 16.2% (n = 4,007) had an infection during hospitalisation (18.0% in those transfused with allogeneic blood; 9.7% in those transfused with autologous blood only; and 6.6% in those not transfused; *P *< 0.001). Receipt of allogeneic blood was associated with infection across various sites (Table 2). There was a statistically significant increase in infections of the genitourinary system, respiratory tract, skin or subcutaneous tissue and digestive tract in those given allogeneic blood, as well as an increase in septicaemia or sepsis, other postoperative infections, and infection with *Clostridium difficile*.

**Table 2:** Association between allogeneic blood transfusion and type of infection during hospitalisation for coronary artery bypass graft surgery

Outcome:	Number (%) with infection during hospitalisation	Odds ratio for allogeneic transfusion* (95% CI)	*P *value
Genitourinary system infection	1,567 (6.3%)	1.27 (1.03, 1.55)	0.023
Respiratory tract infection	1,247 (5.0%)	2.45 (1.88, 3.21)	< 0.001
Septicaemia or sepsis	506 (2.0%)	3.65 (2.22, 6.00)	< 0.001
Infection of skin or subcutaneous tissue	392 (1.6%)	2.27 (1.48, 3.48)	< 0.001
Postoperative infection (site undefined)	382 (1.5%)	2.02 (1.31, 3.11)	0.002
Infection with *C. difficile*	127 (0.5%)	3.16 (1.36, 7.35)	0.008
Circulatory system infection	130 (0.5%)	1.65 (0.92, 2.95)	0.093
Digestive tract infection	118 (0.5%)	2.11 (1.01, 4.44)	0.048

Note: some patients had more than one type of infection.

*Adjusted for propensity score, surgeon volume and hospital volume.

**Figure 2:**
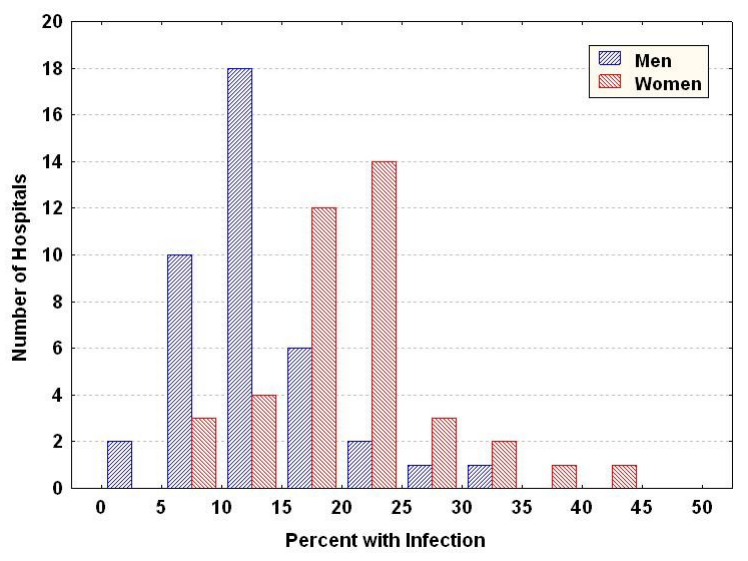
**Variation across hospitals in infection for men and women undergoing coronary artery bypass graft surgery**.

There was also variation in infection rates across the hospitals (Figure 2), with a greater frequency in women than in men (21.1% vs 13.4%, respectively; *P *< 0.001). Hospital infection rates ranged from 3.9% to 34.2% in men and from 7.7% to 43.0% in women.

### Regression models

Odds ratios for the association between transfusion and study outcomes are presented in Table 3. In general, patients who received allogeneic transfusions (n = 20,789) exhibited poorer outcomes than patients who received autologous transfusions only (n = 308). The odds of infection during hospitalisation in patients who received an allogeneic transfusion were twice that of patients who were not transfused. For those receiving allogeneic blood, the odds of death during hospitalisation were elevated nearly fivefold with elective surgeries and nearly fourfold with non-elective surgeries. Likewise, the odds of death in the 30 days after discharge were elevated for elective surgeries nearly threefold and, for non-elective surgeries, over fourfold with allogeneic blood. Readmission within 30 days post discharge was significantly increased in those transfused with allogeneic blood, regardless of type of surgery. Readmission for reason of infection was not statistically significant, although it approached significance for those who received allogeneic blood with either urgent or emergency surgery.

**Table 3:** Odds ratios for the association between transfusion and outcomes for patients who underwent coronary artery bypass graft surgery

Outcomes:	Number (%) with outcome	Odds ratio for autologous transfusion (95% CI)*	*P *value	Odds ratio for allogeneic transfusion (95% CI)*	*P *value
Elective surgery:					
In-hospital infection	1,305 (11.4%)	1.02 (0.56, 1.86)	0.936	1.98 (1.59, 2.46)	< 0.001
In-hospital mortality	325 (2.8%)	0.96 (0.12, 7.69)	0.968	4.67 (2.38, 9.18)	< 0.001
30-day readmission to hospital	2,488 (22.3%)	0.70 (0.44, 1.12)	0.140	1.43 (1.25, 1.65)	< 0.001
30-day readmission for infection	402 (3.6%)	0.94 (0.39, 2.24)	0.881	1.09 (0.82, 1.46)	0.555
30-day post-discharge mortality	155 (1.4%)	1.06 (0.13, 8.69)	0.957	2.88 (1.38, 5.98)	0.005
Urgent or emergency surgery:					
In-hospital infection	2,702 (20.3%)	1.99 (1.12, 3.55)	0.019	1.82 (1.51, 2.20)	< 0.001
In-hospital mortality	637 (4.8%)	1.24 (0.16, 9.68)	0.837	3.82 (2.18, 6.70)	< 0.001
30-day readmission to hospital	3,610 (28.5%)	1.36 (0.79, 2.34)	0.266	1.68 (1.44, 1.96)	< 0.001
30-day readmission for infection	543 (4.3%)	1.88 (0.72, 4.90)	0.199	1.39 (1.00, 1.93)	0.052
30-day post-discharge mortality	290 (2.3%)	NE	NE	4.65 (1.90, 11.39)	0.001

The comparison group consisted of patients who received no transfusions.

*Adjusted for propensity score, surgical volume and hospital volume.

NE = not estimable due to small numbers.

The odds of infection, mortality and readmission were not significantly elevated for patients who received only autologous blood compared to those not receiving any transfusions, with one exception. The odds of in-hospital infection were elevated with patients receiving autologous transfusions with urgent or emergency surgery.

Using the predicted values from the final mixed-effects model with propensity score adjustment, the rate of in-hospital infection could be reduced to 13.1% (with 759 fewer cases in this population) if all hospital transfusion rates were 60%. If the transfusion rate were lowered to 50% at the hospital level, the expected rate of in-hospital infection would be 11.8%, with 1,070 fewer patients infected. For every 1% increase in the rate of transfusion, there was a 0.13% increase in the probability of infection at the hospital level.

### Intraclass correlation coefficients

Intraclass correlation coefficients were calculated and are listed in Table 4. Overall, the proportion of variation in allogeneic transfusion attributable to hospital was 29.95% (95% CI 20.45% to 41.55%). This variation remained similar across types of admission (elective, urgent, emergency), patient age category, and gender. However, the proportion of variation due to hospital was considerably less for African-Americans than for whites (6.47% and 29.97%, respectively). This finding was investigated further at both the patient level and hospital level (Table 5). Patients who received their surgery at African-American hospitals exhibited the greatest frequency of adverse outcomes, regardless of their race.

**Table 4:** Variation is use of allogeneic transfusion attributable to hospital for patients who underwent coronary artery bypass graft surgery

Group:	Intraclass correlation coefficient	95% Confidence interval
Overall	29.95%	20.45% to 41.55%
Type of admission:		
Elective	29.30%	19.67% to 41.22%
Urgent	25.68%	16.35% to 37.91%
Emergency	26.43%	16.14% to 40.14%
Age:		
< 70 years	30.06%	19.85% to 42.71%
≥ 70 years	27.44%	18.26% to 39.04%
Gender:		
Men	31.17%	21.15% to 43.33%
Women	24.77%	15.36% to 37.38%
Race:		
White	29.97%	20.43% to 41.63%
African-American	6.47%	1.89% to 19.86%

**Table 5:** Adverse outcomes by patient-level and hospital-level race

	Percentage transfused	Percentage with infection	In-hospital mortality	30-Day readmission
African-American hospitals:				
African-American patients	93.7%	34.5%	7.0%	51.2%
Other patients	90.0%	26.1%	5.7%	34.1%
Non-African-American hospitals:				
African-American patients	89.5%	19.3%	4.9%	29.8%
Other patients	83.4%	15.7%	3.8%	24.0%

## Discussion

### Variation by hospital

Variability in transfusion practices remains a concern. Overall, 30% of the variability in transfusion practices after CABG surgery was attributable to hospital site. This variation was present regardless of the type of admission, age group or gender of the patient. The overall intraclass correlation coefficient, generated from actual patients in this study, was slightly higher than an estimate calculated from simulated data [10]. In a cross-sectional survey using 8 case simulations sent to physicians in 32 Canadian hospitals in 2004 [10], the intrahospital correlation coefficient for red cell transfusion triggers in CABG surgery was found to be 14.0% to 24.2%, depending upon the case presentation and the time of administration (intraoperative vs postoperative).

Our findings show that there was greater use of allogeneic blood among African-Americans, as well as less variation in transfusion practices across hospitals in such patients. Cross-classifying by patient and hospital levels revealed that the effects of race were particularly important at the hospital level. African-American hospitals had elevated rates of infection, mortality and readmission in patients of all races. African-American patients had better outcomes when they received surgery at non-African-American hospitals. Kim and colleagues also reported a hospital effect on mortality in African-Americans in a study of academic medical centres in the US [11]. They found that receiving CABG surgery at a higher volume hospital was of particular benefit to African-American patients.

There have been previous reports of hospital-wide differences in transfusion practices for patients receiving CABG surgery in the US [12-15]. In a study of 18 US institutions [12], plasma use ranged from 0% to 97%; platelet use ranged from 0% to 80%; and mean allogeneic red blood cell use ranged from 0.4 to 6.3 units across hospitals. In a study of 24 academic institutions using low-risk patients only [13], 27% to 92% of patients were transfused with packed red blood cells at the hospital level; 0% to 36% of patients received platelets; 0% to 36% received fresh-frozen plasma; and 0% to 17% of patients were given cryoprecipitate. In an investigation of 14 Veterans Administration Medical Centers [14], intraoperative transfusion use in hospitals varied from 1.6% to 28.4% for red blood cells, 0% to 9.7% for fresh-frozen plasma and 4.8% to 18.4% for platelets. In a study of 5 university teaching hospitals in the United States [15], transfusion of all blood components ranged from 324 to 1,019 units across hospitals. Such variation persisted in the late 1990s. In a study using 1996 to 2001 data from 32 US hospitals [8], variation remained substantial ranging from 0% to 100% for various blood components.

Mandatory hospital-wide programs to improve transfusion practices have resulted in some success in reducing the use of transfusions [16], as have specific transfusion guidelines in intensive care units [17] and the adoption of transfusion coordinators [18]. Some hospitals have instituted bloodless surgery programs while others have utilised restriction or management policies [19]. For example, Earley and colleagues found that implementation of transfusion restriction practices significantly reduced the incidence of ventilator-associated pneumonia in trauma patients [20]. Evidence-based guidelines for blood conservation techniques have been published with specific recommendations for preoperative and intraoperative measures to reduce blood use in the postoperative period [21]. Such multimodal measures may warrant the coordination of efforts across disciplines within the hospital. In particular, research regarding interventions targeted at hospitalists may be valuable.

### Transfusion and adverse outcomes

Our finding of substantial variation is of particular concern since the receipt of allogeneic blood yields risks as well as benefits. The current study demonstrated increased infection rates for different sites throughout the body suggesting a systemic immunosuppressive effect in the recipient. The increased risk of infection was apparent in the bloodstream, respiratory tract, digestive tract, urinary tract, skin, and non-specific sites. While the most common infections were of the genitourinary system and respiratory tract in our study, the strongest associations were for septicaemia or sepsis and for infections with *Clostridium difficile*, both of which carry higher rates of mortality. Allogeneic blood transfusion is not yet recognised as a risk factor for *C. difficile *infection [22] but our data indicate that this may be an issue for cardiac surgical patients.

Our findings are consistent with previous work in this field. For example, a meta-analysis of 20 prospective studies (1986 to 2000) with 13,152 hospitalised patients, reported a summary odds ratio for the relation between blood transfusion and postoperative bacterial infection of 3.45 overall and 5.26 for trauma patients [23]. Mechanisms underlying these adverse effects include the contributory effects of leukocytes in allogeneic blood, which resulted in widespread leukoreduction of blood components [24,25]. Recent evidence points to possible storage problems with blood. Changes in the red cell storage lesion are time dependent and several studies have shown increasing rates of infection, length of stay, and mortality, as well as acute renal dysfunction in cardiac patients with longer durations of storage [7,26]. Koch and colleagues reported that cardiac patients who received red blood cells that were stored for more than 14 days had significantly higher rates of septicaemia or sepsis and mortality than patients receiving red blood cells stored for a shorter length of time [7].

### Limitations

Our findings should be considered in the context of the following limitations. First, by nature of the observational study design, we cannot rule out unmeasured factors that may account for these outcomes. In particular, we did not measure left ventricular systolic function or left main coronary artery disease, which may influence prognosis after CABG surgery. Other important factors unavailable in this database included procedural details (for example, intra-aortic balloon pump, off-pump surgery), medication use (for example, aprotinin), perioperative haemoglobin concentrations, and length of storage of the transfused blood. Each of these factors could have been differentially distributed in patients at particular hospitals and therefore, may have accounted for the hospital level variation in practices or differences in patient outcomes.

Unfortunately, we could not adequately assess the effects of specific blood components in this study. Although most evidence points to the deleterious effects of red blood cells on rates of nosocomial infection [1-5], there are fewer investigations of the effects of platelets and plasma, and such reports give conflicting results [27-31]. If the association with infection is specific for red blood cells only, the pooling of patients with different blood components in our study would have biased the reported odds ratios towards the null.

In a previous investigation, the sensitivity and specificity of using ICD-9 procedure codes for blood transfusion have been found to be 83% and 100%, respectively [32]. In this study, revenue centre blood codes were also used and therefore, we expect that the sensitivity is greater than using procedure codes alone.

## Conclusion

In summary, we found that 30% of the variation in transfusion practices was attributable to hospital. Allogeneic but not autologous transfusion was associated with nosocomial infection at multiple sites, readmission to the hospital within 30 days after discharge, and mortality. The safety of patients undergoing CABG will likely be improved if hospitals carefully review current guidelines on allogeneic blood transfusion, closely adhere to such guidelines, and institute interventions to reduce inappropriate use of blood transfusions in recipients of CABG.

## Competing interests

The authors declare that they have no competing interests.

## Authors' contributions

MAMR and NB participated in the initial inception of the study hypotheses. MAMR, SS and KML assisted in securing funding for the project. MAMR, SS and KML assisted in obtaining the data. MAMR conducted the statistical analyses. NB, SS, KLM and BKM assisted in directing the analyses. MAMR, NB, SS, KLM and BKN participated in drafting and editing the manuscript. MAMR, NB, SS, KLM and BKN approved the final manuscript.

## Pre-publication history

The pre-publication history for this paper can be accessed here:


